# Twenty-Eighth Annual Report of the Managers of the State Lunatic Asylum

**Published:** 1871-08

**Authors:** 


*Twenty-Eighth Annual Report of the Managers of the State Lunatic Asylum.*

According to this interesting reports, much has been done during the past year to advance the pathological investigations of the cause of insanity, and Dr. E. R. Hun has been appointed special pathologist. The plan proposed by Dr. J. P. Gray, upon which the investigations are conducted, is as follows:

FIRST. The examination of secretions in all stages of the disease.

SECOND. The pulse under the sphygmograph, to determine its force and character, and whether any, and if so, what coincident relations its various phases may bear to physical states and psychological manifestations.

THIRD. The pulse under the sphygmograph to show the influence of medicine on the circulation.

FOURTH. Examination with the ophthalmoscope, to ascertain the relations of morbid changes in the optic nerve, vessels, etc., of the eye, to pathologic conditions of the brain and its membranes.

FIFTH. The skin, its temperature, color, elasticity, sensibility, etc., in the several forms and stages of the disease.

SIXTH. *Post-mortem* appearances, generally and microscopically.

SEVENTH. Photographic representations of morbid conditions and specimens.

